# Reporting randomised trials of social and psychological interventions: the CONSORT-SPI 2018 Extension

**DOI:** 10.1186/s13063-018-2733-1

**Published:** 2018-07-31

**Authors:** Paul Montgomery, Sean Grant, Evan Mayo-Wilson, Geraldine Macdonald, Susan Michie, Sally Hopewell, David Moher, on behalf of the CONSORT-SPI Group, J. Lawrence Aber, Doug Altman, Kamaldeep Bhui, Andrew Booth, David Clark, Peter Craig, Manuel Eisner, Mark W. Fraser, Frances Gardner, Sean Grant, Larry Hedges, Steve Hollon, Sally Hopewell, Robert Kaplan, Peter Kaufmann, Spyros Konstantopoulos, Geraldine Macdonald, Evan Mayo-Wilson, Kenneth McLeroy, Susan Michie, Brian Mittman, David Moher, Paul Montgomery, Arthur Nezu, Lawrence Sherman, Edmund Sonuga-Barke, James Thomas, Gary VandenBos, Elizabeth Waters, Robert West, Joanne Yaffe

**Affiliations:** 1 School of Social Policy, University of Birmingham, Edgbaston, Birmingham, B15 2TT UK; 2 Behavioral & Policy Sciences, RAND Corporation, 1776 Main Street, Santa Monica, 90407-2138 CA USA; 3 Department of Epidemiology, 615 North Wolfe Street, E6036, Baltimore, 21205 MD USA; 4 School for Policy Studies, 8 Priory Road, Bristol, BS8 1TZ UK; 5 Department of Clinical, Educational and Health Psychology, Centre for Behaviour Change, London, WC1E 7HB UK; 6 Oxford Clinical Trials Research Unit, Nuffield Department of Orthopaedics, Rheumatology, and Musculoskeletal Sciences, University of Oxford, Botnar Research Centre, Windmill Road, Oxford, OX3 7LD UK; 7 Centre for Journalology, Clinical Epidemiology Program, Ottawa, K1H 8L6 ON Canada

**Keywords:** CONSORT, Randomised controlled trial, Reporting guideline, Reporting standards, Transparency

## Abstract

**Background:**

Randomised controlled trials (RCTs) are used to evaluate social and psychological interventions and inform policy decisions about them. Accurate, complete, and transparent reports of social and psychological intervention RCTs are essential for understanding their design, conduct, results, and the implications of the findings. However, the reporting of RCTs of social and psychological interventions remains suboptimal. The CONSORT Statement has improved the reporting of RCTs in biomedicine. A similar high-quality guideline is needed for the behavioural and social sciences. Our objective was to develop an official extension of the *Consolidated Standards of Reporting Trials 2010 Statement* (CONSORT 2010) for reporting RCTs of social and psychological interventions: CONSORT-SPI 2018.

**Methods:**

We followed best practices in developing the reporting guideline extension. First, we conducted a systematic review of existing reporting guidelines. We then conducted an online Delphi process including 384 international participants. In March 2014, we held a 3-day consensus meeting of 31 experts to determine the content of a checklist specifically targeting social and psychological intervention RCTs. Experts discussed previous research and methodological issues of particular relevance to social and psychological intervention RCTs. They then voted on proposed modifications or extensions of items from CONSORT 2010.

**Results:**

The CONSORT-SPI 2018 checklist extends 9 of the 25 items from CONSORT 2010: background and objectives, trial design, participants, interventions, statistical methods, participant flow, baseline data, outcomes and estimation, and funding. In addition, participants added a new item related to stakeholder involvement, and they modified aspects of the flow diagram related to participant recruitment and retention.

**Conclusions:**

Authors should use CONSORT-SPI 2018 to improve reporting of their social and psychological intervention RCTs. Journals should revise editorial policies and procedures to require use of reporting guidelines by authors and peer reviewers to produce manuscripts that allow readers to appraise study quality, evaluate the applicability of findings to their contexts, and replicate effective interventions.

## Background

When feasible and appropriate, randomised controlled trials (RCTs) are used to evaluate social and psychological interventions, and to inform policy and practice decisions [1–5]. To use reports of RCTs, readers need information about their design, context, conduct, analysis, results, and interpretation. Like other types of research, RCTs can provide biased estimates of intervention effects if they are not conducted well, and syntheses of these RCTs may be biased if the trials are not reported completely [6, 7]. Consequently, accurate, complete, and transparent reports of RCTs are essential for maximising their value [8], allowing replication studies to build the evidence base [9], and facilitating the comparison and implementation of effective interventions in real-world contexts [10].

Recent reviews have shown that reports of RCTs of social and psychological interventions are often insufficiently accurate, comprehensive, and transparent to replicate trials, assess their quality, and understand for whom and under what circumstances an intervention should be delivered [11–13]. For instance, authors often do not report data on intervention implementation [14], such as the specific techniques employed by intervention providers; adaptation or tailoring of the intervention to specific groups or individuals; materials used to support intervention implementation; and participant behaviours [15]. Inadequate reporting can make it difficult for researchers to replicate trials, for intervention developers to design effective interventions, and for providers to use the interventions in practice [16]. A lack of sharing trial protocols, outcome data, and materials required to implement social and psychological interventions has been identified as a major reason for limitations in the ability of behavioural and social scientists to reproduce trial procedures, replicate trial results, and effectively synthesise evidence on these interventions [16–21]. The review of trials that we conducted in the first phase of this project (*n*  =  239) revealed that many CONSORT items were poorly reported in the behavioural and social science literature. Such items included identification as a randomised trial in titles; information about masking, methods for sequence generation, and allocation concealment; and details about the actual delivery of the interventions. Only 11 of 40 journals we examined referenced reporting guidelines in ‘Instructions to Authors’ [11]. This inefficient use of resources for research likely contributes to the suboptimal dissemination of potentially effective interventions [8, 22], overestimations of intervention efficacy [23], and research waste of investment to the order of hundreds of billions of dollars [22]. As in other areas of research, transparent and detailed reporting of social and psychological intervention RCTs is needed to minimise reporting biases and maximise the credibility and utility of this research evidence [24, 25].

### The CONSORT Statement

To address the problems in scientific manuscripts outlined above, reporting guidelines have been developed that include minimum standards for describing specific types of research [26]. Reporting guidelines do not provide recommendations for study design or conduct. Instead, they focus on *reporting* what was done (methods) and what was found (results). In 1996, a group of scientists and journal editors published the CONSORT (Consolidated Standards of Reporting Trials) Statement to help authors report RCTs in biomedicine completely and transparently [27]. In light of feedback and emerging evidence, the CONSORT Group updated this reporting guideline in 2001 [28] and again in 2010 [29]. CONSORT 2010 includes a 25-item checklist and flow diagram. An extensive *Explanation and Elaboration* (E&E) document serves as a user manual that explains the rationale behind each checklist item, provides the methodological rationale for each checklist item, and gives examples of trial details adequately reported in accordance with each checklist item [26].

The CONSORT Statement has had an important impact in medicine. An early evaluation showed that reporting in the *BMJ*, *Lancet*, and *JAMA* improved after the publication of the first CONSORT Statement [30]. Systematic reviews comparing articles in medical journals endorsing CONSORT compared with journals not endorsing it found that the former are significantly more likely to describe the method of sequence generation, allocation concealment, and participant flow [31]. These effects remain even after controlling for the impact factor of the journals and study outcomes [32]. Over 600 journals and prominent editorial groups (including the International Committee of Medical Journal Editors, the Council of Science Editors, and the World Association of Medical Editors) officially endorse the CONSORT Statement.

### Scope of CONSORT-SPI 2018

The CONSORT 2010 Statement focuses on individually randomised two-group parallel trials [29]. To address the varying amount of additional information needed for different types of trial, the CONSORT Group has created extensions (http://www.consort-statement.org/extensions). These extensions target different types of trial designs, such as cluster randomised [33], noninferiority [34], pragmatic [35], N-of-1 [36], and feasibility [37]; different types of trial data, such as patient-reported outcomes [38], abstracts [39], and harms [40]; and different types of intervention (see next section) [41–43]. Intervention extensions of CONSORT are organised by techniques, such as non-pharmacologic [41], herbal medicinal products [42], and acupuncture [43].

Social and psychological interventions go beyond simply adding techniques or using different techniques compared to biomedical interventions; they often use concepts, theories, and taxonomies that are distinct from those used by the biomedical scientists targeted by the CONSORT extension for non-pharmacologic treatments [21, 44–48]. To delineate the scope of CONSORT for social and psychological interventions (CONSORT-SPI), we define interventions by their mechanisms of action: i.e., how these interventions function to affect desired outcomes [49, 50]. That is, social and psychological interventions are actions intended to modify processes and systems that are social and psychological in nature (such as cognitions, emotions, behaviours, norms, relationships, and salient aspects of the environment) and are hypothesised to be influences on outcomes of interest [51, 52].

Social and psychological interventions can be complex in several ways [12, 50]. For example, these interventions cover an assortment of coordinated actions—such as practices, programmes, and policies—that often involve multiple interacting components. The units targeted by these interventions may include individuals, groups, or even places, and outcomes may be measured at any of these levels. The behaviours of both providers and recipients must be understood if the intervention and its effects are to be understood [53–55]. Social and psychological interventions may not follow strictly standardised implementation procedures [56], and effects may depend on aspects of the hard-to-control dynamic systems in which they occur [57–59]. For these reasons, readers of social and psychological intervention research are interested in more than just effect estimates—they require information about how and why these interventions work, for whom, and under what conditions [60].

## Methods

**Fig. 1:**
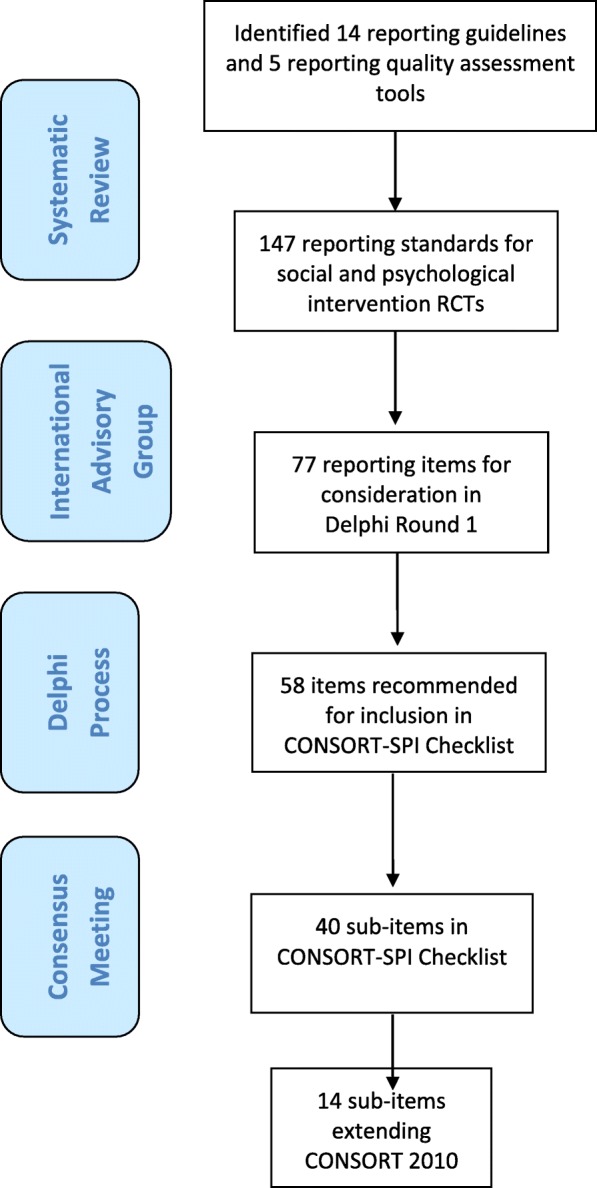
Flow of potential checklist items through CONSORT-SPI 2018 project

We developed an official CONSORT Extension that addresses the *minimum criteria* that need to be met when reporting RCTs evaluating the effects of social and psychological interventions (CONSORT-SPI 2018). We followed recommended practices for developing and disseminating reporting guidelines [26] as described in the study protocol [61]. The methods and results of the systematic review, Delphi process, and consensus meeting followed a pre-specified protocol reported in full elsewhere [11, 61]. We briefly summarise the process below (Fig. 1).

### Systematic reviews

We first conducted a systematic review to assess the adherence of RCTs evaluating social and psychological interventions to existing reporting standards, and to identify potential items for the CONSORT-SPI 2018 checklist and flow diagram [11].

### Online Delphi process

We then conducted an international online Delphi process between September 2013 and February 2014 to prioritise the list of potential items for the CONSORT-SPI 2018 checklist and flow diagram that were identified in the systematic review. To encourage widespread participation, we published commentaries in several journals publishing trial reports in the fields of addiction, criminology, education, adult and child psychology and psychiatry, public health, and social work [11, 62–68], directing readers to a recruitment website where they could register. We also invited members of professional bodies, funders, policymakers, journal editors, practitioners, user representatives, and other stakeholders to participate. We encouraged all identified stakeholders to invite any further colleagues to participate. We sent these participants a two-round survey to rate the importance of including proposed items in the CONSORT-SPI 2018 checklist and to provide qualitative feedback (survey items can be accessed at the project’s ReShare site: 10.5255/UKDA-SN-851981). We synthesised the results of the first survey and sent these to participants, who then completed the second survey, which was designed to explore areas of disagreement and to resolve questions arising during the first round.

### Consensus meeting

Following the Delphi process, we held a three-day in-person consensus meeting to determine the content of the CONSORT-SPI 2018 checklist and flow diagram, as well as the accompanying E&E document (March 2014). We used established methods [69] from previous CONSORT meetings [29, 35, 41, 70]. Participants included 31 experts from the Delphi process (see Table 6 in the Appendix), whom we selected purposively to include key stakeholders from targeted disciplines (e.g. public health, social work, education, criminology, and clinical psychology) and professional roles (e.g. trialists, funders, and journal editors) [71].

Prior to the meeting, we sent participants background literature [9, 11, 26, 39, 61, 64, 72], results from the Delphi process, and the meeting agenda. On the first day, participants discussed the background literature and its applicability to the various disciplines and professional roles represented at the meeting. During the second day, participants discussed and voted on potential checklist and flow diagram items nominated during the Delphi process using anonymous electronic ballots. On the third day, participants voted on the remaining items and discussed strategies for dissemination. Participants were asked to consider the value of each item based on the evidence presented and to vote on whether each item was essential when reporting *all* social and psychological intervention RCTs. When voting, participants could select ‘exclude’, ‘include’, or ‘unsure’.

In the first round of voting, only items endorsed as ‘include’ by ≥70% of participants were included in the checklist [73, 74]. We excluded all other items unless at least two participants proposed they be reconsidered. In this second round of voting, items endorsed as ‘include’ by ≥80% of participants were also incorporated in the CONSORT-SPI 2018 checklist. Participants suggested that several ‘excluded’ items should be discussed in the E&E document.

### Post-meeting activities

After the consensus meeting, we finalised the CONSORT-SPI 2018 checklist and flow diagram. We then drafted the Extension Statement (this manuscript), as well as an E&E document that serves as a user manual for the checklist. We distributed these documents to consensus meeting participants for feedback and revision, and we incorporated their comments in the final version of this manuscript and the accompanying E&E. We also discussed how best to optimise our strategy for disseminating and implementing these documents.

## Results

### Systematic review

The systematic review of reporting guidance identified 14 relevant reporting guidelines and 5 reporting assessment tools. These tools included a total of 147 potential items to consider for the CONSORT-SPI 2018 checklist, 89 of which were not included in the CONSORT checklist [11].

### Online Delphi process

With input from the project’s International Advisory Group, we included 77 potential checklist items from the systematic review in the first round of the modified Delphi process. We recruited 384 Delphi participants from 32 countries working in over a dozen areas of social and psychological intervention, including academics, researchers, practitioners, journal editors, research funders, policymakers, and recipients of social and psychological interventions. The Delphi process yielded 58 potential items as important to consider for inclusion in the CONSORT-SPI 2018 checklist.

### Consensus meeting

**Fig. 2:**
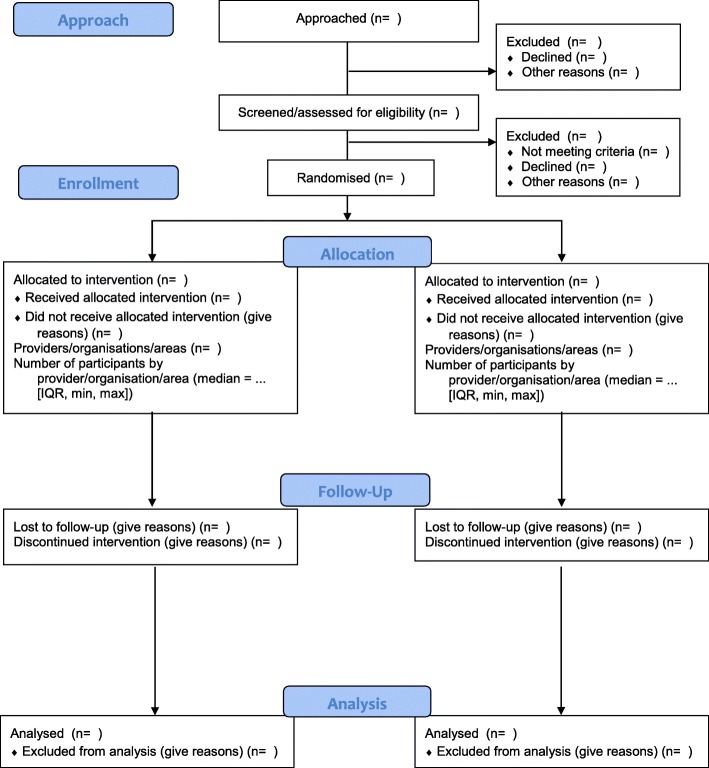
The CONSORT-SPI 2018 flow diagram

During the consensus meeting, participants voted to extend 9 of the 25 items in the CONSORT 2010 checklist: background and objectives, trial design, participants, interventions, statistical methods, participant flow, baseline data, outcomes and estimation, and funding. These extended checklist items addressed the need for reports of RCTs of social and psychological interventions to describe: the hypotheses for how the intervention might work, the eligibility criteria for settings and providers, the actual provider delivery and participant uptake of the interventions, the intervention materials, how missing data were handled, participant recruitment, socioeconomic baseline variables, availability of trial data, author declarations of interest, involvement of the intervention developer in the trial, and details of any incentives offered (Table 1). Participants also voted to add a new item about stakeholder involvement, and they recommended modifications to existing CONSORT 2010 checklist items (Table 2). The flow diagram (Fig. 2) to address the unique needs of social and psychological intervention trials was also modified—specifically, the number of participants approached during enrolment and the number of providers, organisations, and areas (as appropriate) allocated to each trial arm. To further facilitate use of CONSORT-SPI 2018, we have provided a tailored CONSORT Extension for Abstracts (Table 3) [39] and a CONSORT Extension for Cluster Randomised Trials (Tables 4 and 5) [33] for social and psychological intervention trials.

**Table 1:** The CONSORT-SPI 2018 checklist

Section	Item #	CONSORT 2010	CONSORT-SPI 2018
Title and abstract			
	1a	Identification as a randomised trial in the title^§^	
	1b	Structured summary of trial design, methods, results, and conclusions (for specific guidance see CONSORT for Abstracts)^§^	Refer to CONSORT extension for social and psychological intervention trial abstracts
Introduction			
Background and objectives	2a	Scientific background and explanation of rationale^§^	
	2b	Specific objectives or hypotheses^§^	If pre-specified, how the intervention was hypothesised to work
Methods			
Trial design	3a	Description of trial design (such as parallel, factorial), including allocation ratio^§^	If the unit of random assignment is not the individual, please refer to CONSORT for Cluster Randomised Trials [33]
	3b	Important changes to methods after trial commencement (such as eligibility criteria), with reasons	
Participants	4a	Eligibility criteria for participants^§^	When applicable, eligibility criteria for settings and those delivering the interventions
	4b	Settings and locations where the data were collected	
Interventions	5	The interventions for each group with sufficient details to allow replication, including how and when they were actually administered^§^	
	5a		Extent to which interventions were actually delivered by providers and taken up by participants as planned
	5b		Where other informational materials about delivering the intervention can be accessed
	5c		When applicable, how intervention providers were assigned to each group
Outcomes	6a	Completely defined pre-specified outcomes, including how and when they were assessed^§^	
	6b	Any changes to trial outcomes after the trial commenced, with reasons	
Sample size	7a	How sample size was determined^§^	
	7b	When applicable, explanation of any interim analyses and stopping guidelines	
Randomisation			
Sequence generation	8a	Method used to generate the random allocation sequence	
	8b	Type of randomisation; details of any restriction (such as blocking and block size)^§^	
Allocation concealment mechanism	9	Mechanism used to implement the random allocation sequence, describing any steps taken to conceal the sequence until interventions were assigned^§^	
Implementation	10	Who generated the random allocation sequence, who enrolled participants, and who assigned participants to interventions^§^	
Awareness of assignment	11a	Who was aware of intervention assignment after allocation (for example, participants, providers, those assessing outcomes), and how any masking was done	
	11b	If relevant, description of the similarity of interventions	
Analytical methods	12a	Statistical methods used to compare group outcomes^§^	How missing data were handled, with details of any imputation method
	12b	Methods for additional analyses, such as subgroup analyses, adjusted analyses, and process evaluations	
Results			
Participant flow (a diagram is strongly recommended)	13a	For each group, the numbers randomly assigned, receiving the intended intervention, and analysed for the outcomes^§^	Where possible, the number approached, screened, and eligible prior to random assignment, with reasons for non-enrolment
	13b	For each group, losses and exclusions after randomisation, together with reasons^§^	
Recruitment	14a	Dates defining the periods of recruitment and follow-up	
	14b	Why the trial ended or was stopped	
Baseline data	15	A table showing baseline characteristics for each group^§^	Include socioeconomic variables where applicable
Numbers analysed	16	For each group, number included in each analysis and whether the analysis was by original assigned groups^§^	
Outcomes and estimation	17a	For each outcome, results for each group, and the estimated effect size and its precision (such as 95% confidence interval)^§^	Indicate availability of trial data
	17b	For binary outcomes, the presentation of both absolute and relative effect sizes is recommended	
Ancillary analyses	18	Results of any other analyses performed, including subgroup analyses, adjusted analyses, and process evaluations, distinguishing pre-specified from exploratory	
Harms	19	All important harms or unintended effects in each group (for specific guidance see CONSORT for Harms)	
Discussion			
Limitations	20	Trial limitations, addressing sources of potential bias, imprecision, and, if relevant, multiplicity of analyses	
Generalisability	21	Generalisability (external validity, applicability) of the trial findings^§^	
Interpretation	22	Interpretation consistent with results, balancing benefits and harms, and considering other relevant evidence	
Important information			
Registration	23	Registration number and name of trial registry	
Protocol	24	Where the full trial protocol can be accessed, if available	
Declaration of interests	25	Sources of funding and other support; role of funders	Declaration of any other potential interests
Stakeholder involvement*	26a		Any involvement of the intervention developer in the design, conduct, analysis, or reporting of the trial
	26b		Other stakeholder involvement in trial design, conduct, or analyses
	26c		Incentives offered as part of the trial

This table lists items from the CONSORT 2010 checklist (with some modifications for social and psychological intervention trials as described in Table 2) and additional items in the CONSORT-SPI 2018 extension. Empty rows in the ‘CONSORT-SPI 2018’ column indicate that there is no extension to the CONSORT 2010 item

*We strongly recommended that the CONSORT-SPI 2018 Explanation and Elaboration (E&E) document be reviewed when using the CONSORT-SPI 2018 checklist for important clarifications on each item

§An extension item for cluster trials exists for this CONSORT 2010 item

**Table 2:** Noteworthy changes to CONSORT 2010 items in the CONSORT-SPI 2018 checklist

• Item 6a. The distinction between ‘primary’ versus ‘secondary’ outcomes has been removed. • Item 11. ‘Blinding’ has been changed to ‘Awareness of assignment’ and ‘masking’ in the section heading and item wording, respectively. These changes address concerns about the use of the term ‘blinding’ as well as the need to emphasise the issue of awareness of assignment by providers and participants in social and psychological intervention trials. • Item 12. The section heading ‘Statistical methods’ has been changed to ‘Analytical methods’ because some methods may be qualitative in social and psychological intervention RCTs. • Item 12a. The distinction between ‘primary’ versus ‘secondary’ outcomes has been removed. • Item 12b. Process evaluations are specifically highlighted. • Item 13a. The distinction between ‘primary’ versus ‘secondary’ outcomes has been removed. • Items 13a and 16. The wording ‘number of participants’ has been changed to ‘number’ because the term ‘participants’ is not appropriate for RCTs in which the unit of intervention is a geographic area. While social and psychological interventions may target individual participants or groups of individuals, such as families or schools, they may also involve place-based techniques that target geographic units and examine area-level effects. However, for convenience and consistency with the CONSORT 2010 guidance [72], the CONSORT-SPI 2018 checklist and E&E will refer to the unit targeted by the intervention as ‘participants’, though ‘participants’ throughout this guidance is meant to stand for ‘participating units’ or the unit being targeted by the intervention [87], which may include geographic units. • Item 15. The words ‘clinical and demographic’ have been removed because this checklist targets interventions that may not be medical in nature or have health outcomes, and thus to emphasise the need to report important baseline characteristics irrespective of their nature. • Item 16. The parenthetical ‘(denominator)’ has been removed. The term implied the use of dichotomous outcomes, whereas continuous outcomes are extremely prevalent in social and psychological intervention RCTs. • Item 17a. The distinction between ‘primary’ versus ‘secondary’ outcomes has been removed. • Items 23–25. The section ‘Other Information’ has been changed to ‘Important Information’ because consensus meeting participants had concerns that ‘Other’ makes the requested information appear to be of secondary importance to previous sections. • Item 25. The phrase ‘such as supply of drugs’ has been removed because drug trials are not in the purview of this extension by definition. • Item 26: New item. A new sub-section in ‘Important Information’ called ‘Stakeholder Involvement’ has been added because consensus meeting participants thought such a sub-section would best fit the three sub-items currently allocated to it.

**Table 3:** Items to report in journal or conference abstracts for social and psychological intervention trials [39]

Section	CONSORT abstract item	Relevant CONSORT-SPI item
Title	Identification of the study as randomised	
Authors	Contact details for the corresponding author	
Trial design	Description of the trial design (e.g. parallel, cluster, noninferiority)	If the unit of random assignment is not the individual, refer to CONSORT for Cluster Randomised Trials and report the items included in its extension for abstracts [33]
Methods		
Participants	Eligibility criteria for participants and the settings where the data were collected	When applicable, the eligibility criteria for the setting of the intervention delivery and the eligibility criteria for the persons who delivered the interventions
Interventions	Interventions intended for each group	
Objective	Specific objective or hypothesis	If pre-specified, how the intervention was hypothesised to work
Outcomes	Clearly defined primary outcome for this report	
Randomisation	How participants were allocated to interventions	
Awareness of assignment	Who was aware of intervention assignment after allocation (for example, participants, providers, those assessing outcomes), and how any masking was done	
Results		
Number randomly assigned	Number randomised to each group	
Recruitment	Trial status	
Interventions		Extent to which interventions were actually delivered by providers and taken up by participants as planned
Number analysed	Number analysed in each group	
Outcomes	For the primary outcome, a result for each group and the estimated effect size and its precision	
Harms	Important adverse events or side effects	
Conclusions	General interpretation of the results	
Trial registration	Registration number and name of trial register	
Funding	Source of funding

**Table 4:** Items to report in the abstract for cluster randomised social and psychological intervention trials [33]

Section	CONSORT Abstract item	Relevant CONSORT Cluster extension item
Title	Identification of the study as randomised	Identification of study as cluster randomised
Authors	Contact details for the corresponding author	
Trial design	Description of the trial design (e.g. parallel, cluster, noninferiority)	
Methods		
Participants	Eligibility criteria for participants and the settings where the data were collected	Eligibility criteria for clusters
Interventions	Interventions intended for each group	
Objective	Specific objective or hypothesis	Whether objective or hypothesis pertains to the cluster level, the individual participant level, or both
Outcomes	Clearly defined primary outcome for this report	Whether the primary outcome pertains to the cluster level, the individual participant level, or both
Randomisation	How participants were allocated to interventions	How clusters were allocated to interventions
Awareness of assignment	Who was aware of intervention assignment after allocation (for example, participants, providers, those assessing outcomes), and how any masking was done	
Results		
Number randomly assigned	Number of participants randomised to each group	Number of clusters randomised to each group
Recruitment	Trial status	
Number analysed	Number of participants analysed in each group	Number of clusters analysed in each group
Outcomes	For the primary outcome, a result for each group and the estimated effect size and its precision	Results at the cluster or individual level as applicable for each primary outcome
Harms	Important adverse events or side effects	
Conclusions	General interpretation of the results	
Trial registration	Registration number and name of trial register	
Funding	Source of funding

**Table 5:** Items to report in the main text for cluster randomised social and psychological intervention trials [33]

Section	Item #	Cluster extension item
Title	1a	Identification as a cluster randomised trial in the title
Abstract	1b	See Table 4
Introduction		
Background and objectives	2a	Rationale for using a cluster design
	2b	Whether objectives pertain to the cluster level, the individual participant level, or both
Methods		
Trial design	3a	Definition of cluster and description of how the design features apply to the clusters
Participants	4a	Eligibility criteria for clusters
Interventions	5	Whether interventions pertain to the cluster level, the individual participant level, or both
Outcomes	6a	Whether outcome measures pertain to the cluster level, the individual participant level, or both
Sample size	7a	Method of calculation, number of clusters(s) (and whether equal or unequal cluster sizes are assumed), cluster size, a coefficient of intracluster correlation (ICC or *k*), and an indication of its uncertainty
Randomisation		
Sequence generation	8b	Details of stratification or matching if used
Allocation concealment mechanism	9	Specification that allocation was based on clusters rather than individuals and whether allocation concealment (if any) was at the cluster level, the individual participant level, or both
Implementation	10a	Who generated the random allocation sequence, who enrolled clusters, and who assigned clusters to interventions
	10b	Mechanism by which individual participants were included in clusters for the purposes of the trial (such as complete enumeration, random sampling)
	10c	From whom consent was sought (representatives of the cluster, individual cluster members, or both) and whether consent was sought before or after randomisation
Analytical methods	12a	How clustering was taken into account
Results		
Participant flow (a diagram is strongly recommended)	13a	For each group, the numbers of clusters that were randomly assigned, received the intended treatment, and were analysed for the primary outcome
	13b	For each group, losses and exclusions for both clusters and individual cluster members
Baseline data	15	Baseline characteristics for the individual and cluster levels as applicable for each group
Numbers analysed	16	For each group, the number of clusters included in each analysis
Outcomes and estimation	17a	Results at the individual or cluster level as applicable and a coefficient of intracluster correlation (ICC or *k*) for each primary outcome
Generalisability	21	Generalisability to clusters or individual participants (as relevant)

## Discussion

The CONSORT-SPI 2018 Extension is designed to assist authors in writing reports of social and psychological intervention RCTs and to assist peer reviewers and editors in assessing these manuscripts. While we recommend that authors report items in the checklist in the relevant manuscript section (i.e., introduction, methods, results, or discussion), the format of an article will depend on journal style, editorial decisions, expectations within a particular research area, and author discretion. At a minimum, authors should address each checklist item somewhere in the article with the appropriate level of detail and clarity. We recommend subheadings within major sections—particularly the methods and results sections—to help ease of reading. The accompanying CONSORT-SPI 2018 E&E document is a user manual for the CONSORT-SPI 2018 checklist, providing a concise rationale for and description of how best to adhere to each checklist item. We recommend that authors preparing reports of social and psychological intervention RCTs consult the CONSORT-SPI 2018 E&E document when using the CONSORT-SPI 2018 checklist.

This guideline may prove useful to several different stakeholders [75]. Researchers can use CONSORT-SPI 2018, along with the SPIRIT Statement, during trial design to ensure they consider the essential study aspects they will have to describe in future manuscripts. Use of CONSORT-SPI 2018 throughout a trial (from design to reporting) can help improve the accuracy, completeness, and transparency of the final manuscript. Journal editors can enforce policies and procedures to ensure that CONSORT-SPI 2018 is actually used by authors, editors and peer reviewers to improve the social and psychological RCT manuscripts they publish [76]. Research funders who adopt CONSORT-SPI 2018 and other reporting guidelines may receive higher quality grant applications, as well as facilitate the commissioning of the most important and rigorous studies while helping to reduce research waste. Policymakers, practitioners, and systematic reviewers who encourage researchers to use CONSORT-SPI 2018 may find this leads to higher quality publications, which these stakeholders can then use to identify and implement effective interventions for populations and settings of interest. In addition, faculty could use reporting guidelines to train the next generation of researchers, peer reviewers, and journal editors [77].

In highlighting prospective trial registration [78], the publication of protocols [79], and increased sharing of trial data [16, 80], all of which are uncommon in social and psychological intervention research, CONSORT-SPI 2018 also complements other efforts to improve research transparency. Examples of such efforts include the Template for Intervention Description and Replication (TIDieR) checklist (which will replace CONSORT 2010 Item 5) [9], the Behaviour Change Technique taxonomy [21, 44], the Berkeley Initiative for Transparency in the Social Sciences [81], the Data Access and Research Transparency Statement [82], the Center for Open Science [19], the Transparency and Openness Promotion guidelines [16], and the Human Behaviour-Change Project [83].

### Strengths and limitations

We followed recommended best practices in the development of these reporting guidelines and advocate their use to future reporting guideline developers [26]. A challenge that we experienced, and which other reporting guideline developers have faced [84], was the large number of potential checklist items that participants considered to be important for a CONSORT-SPI 2018 guideline. As with the CONSORT 2010 Statement, CONSORT-SPI 2018 represents a set of *minimum* reporting criteria and does not preclude individual authors from addressing other issues that they deem important to ensure complete and transparent reporting. For example, for social and psychological interventions utilising mobile phones, additional details may need to be reported in trial manuscripts [85].

In addition, as in the development of previous CONSORT guidelines, other items fundamental to an RCT have not been included (such as approval by an institutional ethical review board) because journals and institutions address these issues in other ways [29]. We encourage users of this guideline to provide feedback on the appropriateness of the content in the CONSORT-SPI 2018 checklist and its accompanying E&E document.

### Endorsement

As a recognised extension of the CONSORT 2010 Statement, journals and organisations already endorsing the CONSORT guidelines can easily extend their support to CONSORT-SPI 2018. We encourage other journals and organisations that publish social and psychological intervention RCTs to endorse CONSORT-SPI 2018 and to register their official support on the CONSORT website (http://www.consort-statement.org/about-consort/endorsement). Journal endorsement policies that include monitoring of adherence to the checklist are essential for complete and transparent reporting [31]. To maximise the potential impact of CONSORT-SPI 2018, editors should consider requiring authors to submit a completed CONSORT-SPI 2018 checklist as a separate document when reporting social and psychological intervention RCTs, and we recommend that editors should check that all items have been included before sending manuscripts for peer review. Endorsing journals should consider adding the following statement to their ‘Instructions to Authors’ [36]:

*JOURNAL NAME* requires a completed CONSORT-SPI 2018 checklist as a condition for submitting manuscripts about randomised trials of social and psychological interventions. We recommend that your submission addresses each item in the CONSORT-SPI 2018 checklist. Taking the time to ensure your manuscript meets these basic reporting requirements will greatly improve your manuscript, and may potentially enhance its chances for eventual publication.

We also recommend that researchers, editors, peer reviewers, funders, and educators consult the CONSORT website (http://www.consort-statement.org) for other relevant CONSORT Extensions (e.g. the extension for cluster randomised trials) [33], as well as the Enhancing the Quality and Transparency of Health Research (EQUATOR) Network for up-to-date information on other reporting guidelines (http://www.equator-network.org) that may be of relevance to their study.

## Conclusion

CONSORT-SPI 2018, like other CONSORT guidelines, is an evolving tool that requires regular reappraisal and modifications as new evidence emerges and as scientific consensus changes. We invite interested stakeholders to contact us with feedback or to contribute to the guideline’s ongoing development, including individuals or groups who wish to translate the CONSORT-SPI 2018 checklist into other languages or those who wish to evaluate the impact of the CONSORT-SPI 2018 checklist on future trial reporting [31, 86]. To provide feedback and access the most recent version of the CONSORT-SPI 2018 checklist and E&E document, visit the project (https://www.birmingham.ac.uk/schools/social-policy/departments/social-policy-sociology-criminology/research/projects/2017/Consort-SPI.aspx) and CONSORT websites (http://www.consort-statement.org).

## Appendix

Table 6 lists the members of the CONSORT-SPI Group who were participated to represent the stakeholder groups in the consensus meeting.

**Table 6:** The CONSORT-SPI Group

Member	Organisation
Project Executive	
Sean Grant	RAND Corporation
Sally Hopewell	University of Oxford
Evan Mayo-Wilson	Johns Hopkins University
Susan Michie	University College London
David Moher	Ottawa Health Research Institute
Paul Montgomery	University of Birmingham
Geraldine Macdonald	University of Bristol
International Advisory Board	
Stakeholder Representatives of Behavioural and Social Science Disciplines	
J. Lawrence Aber	New York University
David Clark	University of Oxford
Manuel Eisner	University of Cambridge
Frances Gardner	University of Oxford
Steve Hollon	Vanderbilt University
Lawrence Sherman	University of Cambridge
James Thomas	UCL Institute of Education
Elizabeth Waters (deceased)	University of Melbourne
Joanne Yaffe	University of Utah
Stakeholder Representatives of Intervention Research Methodologists	
Andrew Booth	University of Sheffield
Peter Craig	University of Glasgow
Larry Hedges	Northwestern University
Stakeholder Representatives of Journals	
Doug Altman	*Trials*
Mark W. Fraser	*Journal of the Society for Social Work and Research*
Spyros Konstantopoulos	*Journal of Research on Educational Effectiveness*
Kenneth McLeroy	*American Journal of Public Health*
Arthur Nezu	*Journal of Consulting and Clinical Psychology*
Edmund Sonuga-Barke	*Journal of Child Psychology and Psychiatry*
Gary VandenBos	*American Psychologist*
Robert West	*Addiction*
Stakeholder Representatives of Research Funders	
Robert Kaplan	Office of Behavioural and Social Sciences Research
Peter Kaufmann	National Heart, Lung, and Blood Institute
Brian Mittman	Patient-Centered Outcomes Research Institute
